# Characterizing the pathogenicity of genetic variants: the consequences of context

**DOI:** 10.1038/s41525-023-00386-5

**Published:** 2024-01-09

**Authors:** Timothy H. Ciesielski, Giorgio Sirugo, Sudha K. Iyengar, Scott M. Williams

**Affiliations:** 1 The Department of Population and Quantitative Health Sciences at Case Western Reserve University School of Medicine, Cleveland, OH USA; 2 Mary Ann Swetland Center for Environmental Health at Case Western Reserve University School of Medicine, Cleveland, OH USA; 3 Ronin Institute, Montclair, NJ USA; 4 Institute of Systems Pharmacology and Translational Therapeutics, Perelman School of Medicine, University of Pennsylvania, Philadelphia, PA USA; 5 Division of Translational Medicine and Human Genetics, Perelman School of Medicine, University of Pennsylvania, Philadelphia, PA USA; 6 The Department of Genetics and Genome Sciences at Case Western Reserve University School of Medicine, Cleveland, OH USA; 7 Cleveland Institute for Computational Biology, Cleveland, OH USA

**Keywords:** Genetics research, Molecular medicine

## Abstract

Beyond initial discovery of a pathogenic variant, establishing that a variant is recurrently associated with disease is important for understanding clinical impact and disease etiology. Disappointingly, our ability to characterize pathogenicity under varied circumstances is limited. Here we discuss the role of genetic and environmental background and how it affects variant penetrance and outcomes. Specifically, genetic and environmental settings determine penetrance, and we should expect lower penetrance where contexts are diverse. For example, when over 5000 ClinVar pathogenic and loss-of-function variants were assessed in two large biobanks, UK Biobank and BioMe, the mean penetrance was only 7%. This indicates that the participants in the family-based, clinical, and case-control studies that identified these variants were more homogenous and enriched for etiologic co-factors, and the winner’s curse was at play. We also emphasize that the outcome of interest can vary across conditions. The variant that causes hemoglobin S can increase the risk of death from sickling, lower the risk of death from malaria, or increase the risk of kidney disease, depending on the presence of other variants, the endemicity of malaria, and a suite of other factors. Overall, annotation on a single continuum from benign to pathogenic attempts to shoehorn a complex phenomenon into an overly simplistic framework. Variant effects often vary by context, and thus it is critical to assess potential pathogenicity in different settings. There is no panacea or easy fix, but we offer two recommendations for consideration. First, we need to routinely evaluate contexts such as sex and genetic ancestry by conducting stratified analyses and developing methods that can detect heterogenous effects (e.g. female-to-male allele proportion ratios). Second, we need to consistently document what we know about effect modifiers in our annotation databases. These are not the only possible approaches, but they begin to provide means to create robust annotations of pathogenicity.

When we talk about the pathogenicity of genetic variants, what exactly are we talking about? Although this question on its surface may appear to be a trivial or simply philosophical question, it is not. It shapes the foundational logic of human genetics research and determines the utility of our work with respect to disease risk and clinical intervention. In brief, our standard definitions of pathogenicity refer to variants that are deleterious, harmful, or increase the probability of disease^1^. This sounds simple, but it is too simple, as this definition often leads us to ignore a key principle: Genes evolve and function in the contexts created by their environment, including other genetic variants. These contexts can determine penetrance and thus the ability of a variant to cause disease.

## Variant pathogenicity often depends on context

**Fig. 1:**
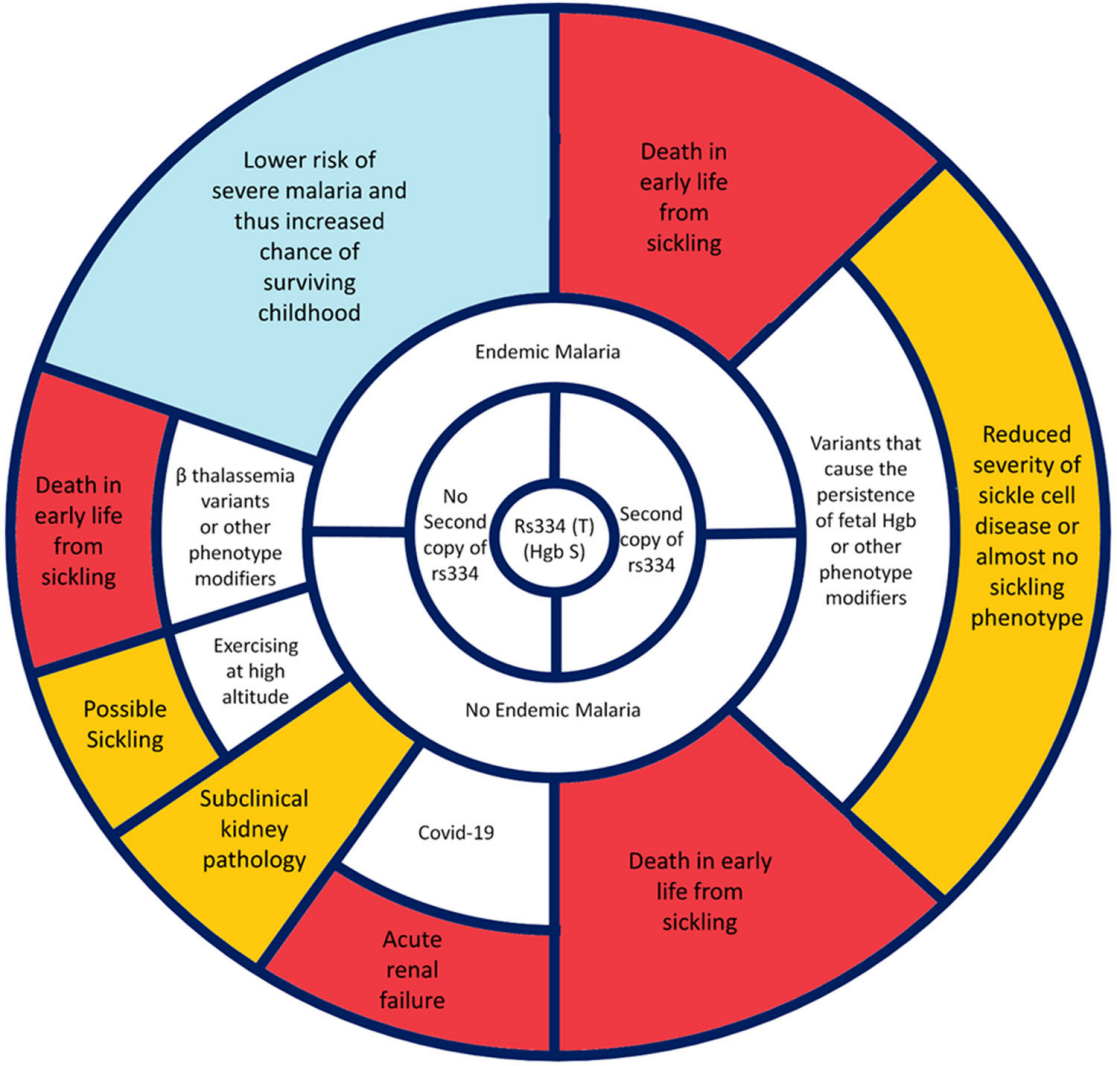
The complex determinants of phenotype and pathogenicity: example of a relatively simple case—Hemoglobin S (rs334). Starting at the center of this schematic and moving out radially in any direction, different relevant contexts are encountered. These contexts determine the type and severity of the observed phenotypes. This schematic is based on our current understanding and is not intended to be an exhaustive description of all relevant and all possible phenotypes linked to rs334. Some of the modifying contexts and relevant phenotypes may yet to be discovered. Finally, although it cannot be comprehensively depicted on this figure, phenotypes may serve as competing risks for one another, and this becomes more complex with age. As an example, a person cannot develop chronic kidney disease in older age if they die of sickling complications at a younger age.

A simple but informative example of the heterogeneity of pathogenicity is the beta globin variant that causes hemoglobin S (HbS). The HbS allele in an individual who is homozygous for this variant has sickle cell disease, thereby increasing risk of death at a young age^2,3^. However, this same allele in the context of a second allele that encodes HbA will reduce the risk of risk of death at a young age in malaria endemic regions^4,5^. This decreased risk of death is the reason that the HbS allele is common in malaria endemic regions, and has not been culled by evolution^6,7^. Furthermore, in regions without malaria, being heterozygous for the HbS allele may not affect risk of death at a young age, unless there exists another precipitating variant in that individual’s genome, or the carrier experiences hypoxia when exercising at high altitude^8–10^. In these distinct, yet malaria-free, contexts, the HbS variant may again increase death risk at a young age. To add to this complexity, data now indicate that older heterozygotes may have an increased risk of subclinical kidney pathology, and increased rates of acute renal failure when exposed to Sars-CoV-2^11^. Finally, variants that decrease the expression of alpha globin subunits (HBA1 and HBA2—alpha thalassemia)^12,13^ or allow for the persistent expression of gamma globin subunits into adulthood (HBG1 and HBG2 – persistence of fetal hemoglobin)^14^ can greatly mitigate the risk of death due to HbS homozygosity. Thus, the pathogenicity of the HbS variant depends heavily on other alleles, the environment, and the health outcome being evaluated. HbS can be considered a “simple” case, but even in this situation, pathogenic potential is strongly shaped by multiple contextual factors (Fig. 1).

This example clarifies that the process of making a universal pathogenicity assessment, uses an oversimplistic framework to describe an inherently complex phenomenon. Even when a variant can cause disease, it often does not, and knowing the modifying factors is critical to evaluating pathogenicity. Thus assuming that genetic variants have a single unidirectional effect on one outcome, obscures the complex genetic architecture of disease^15^. Regulatory processes, genetic buffering, environmental interactions, and epistasis can all play roles in determining the impact of a given variant^16–19^, and these contexts cannot be ignored if we want to understand variant pathogenicity^15^.

## Defining pathogenicity is especially hard for variants with low penetrance and variable expressivity

Nonetheless, attempts are still made to produce “universal pathogenicity” assessments^20^. These assessments may make sense in the context of highly penetrant variants that cause Mendelian disease, but what about low penetrance variants with variable expressivity? Allelic expression levels, epigenetic changes, cis variants, trans variants, environmental exposures, and other factors, including lifestyle, collectively shape variant impact^21,22^ and low penetrance variants make up a very large proportion of our annotations. When over 5000 pathogenic and loss-of-function variants were assessed in the UK Biobank and BioMe, the mean penetrance was unexpectedly low (6.9%, 95% CI: 6.0–7.8%)^23^. While some of this pattern can be partly explained by the factors that drive the winner’s curse (i.e. inflated magnitude of initial associations due to low power, publication bias, model overfitting, etc.)^24,25^, it must be added that smaller associations should be expected when the study participants are more diverse. Family-based, clinical, and case-control studies have more homogenous participants and because study entry is partly conditioned on disease status, these study groups are enriched for etiologic co-factors. This means lower penetrance and smaller effect sizes will often be observed in large population-based cohorts^22,26,27^, even when there are subgroups where penetrance is high. When a variant has a smaller effect size and reduced penetrance in a heterogenous, population-based sample, it is important to examine that variant in multiple contexts. This can identify potentially sensitive subgroups, such as an ancestries, environments, or multiplexed families with higher penetrance and pathogenicity. Overall, assessment of variants in multiple contexts^28,29^ is critical to understanding differences in the causal mechanisms of disease in distinct groups.

## Downplaying this heterogeneity impairs clinical communication and practice

Regardless of the reason for low penetrance, it creates a problem for pathogenicity assessments and clinical genetic practice. When these annotations are used as screening tests for disease risk, there is a systematic problem with test specificity (i.e., the ability of a test to identify true negatives and avoid false positives^30^). Since penetrance among many pathogenic variants is often low, most people with these variants will not develop disease. Thus, when applied clinically this can result in a very large number of false positives and subsequent unnecessary actions. While a strong argument can be made for tolerating false positives (type 1 error) in the early stages of genetic discovery research^31,32^, false positives in clinical settings can lead to patient anxiety, needless expense, and harm^33^.

One way to vet putative pathogenicity is to perform experiments that biologically validate the effects of genetic variants. However, it should be noted that such experiments are limited in their generalizability, and they are restricted by the conditions under which the experiments are performed. In vitro experiments and animal models can clearly demonstrate causal and mechanistic evidence of pathogenicity, but they cannot test or create all relevant contexts. For example, the experimental temperature, day night cycle, diet, air quality, or hormonal milieu may not reflect those of the humans that carry a potentially pathogenic variant. Geneticists are aware of these dynamics, known as *reaction norms*, and they have been taught in genetics classes for decades^34,35^. However some physicians and the general public may not be as familiar with how this fundamental principle of genetic variation can affect our annotations.

Universal pathogenicity assessments also create a systematic problem with sensitivity (i.e., the ability of a test to identify true positives and avoid false negatives^30^). This is partly because our annotation guidelines^36^, even when thoughtfully refined^37^ have traditionally considered the “absence of evidence” to be “evidence of absence”. In other words, when a variant is observed in a high number of healthy people (e.g., minor allele frequency [MAF] >5%) and it has not been yet linked to disease, then it can be labeled benign. Unfortunately, this approach fails to account for the determinants of penetrance. If a key determinant of penetrance was not present among the observations, then a conditionally pathogenic variant can be labeled a *Variant of Unknown Significance* or even *Benign*. This creates many issues but it seems particularly troublesome in the clinic when sequencing patients to identify the cause of rare syndromes^38^. Imagine trying to annotate the phenylalanine hydroxylase gene variants that cause phenylketonuria^39^ in a population with almost no access to foods that contain phenylalanine. Phenylalanine hydroxylase variants would appear benign in this context. Hence, in most cases when variant pathogenicity is assessed, the process identifies what *can* cause disease, but importantly, it does not identify what *will* cause disease in a given person at a given time^40,41^. This context agnostic approach has utility, but its limitations must be acknowledged and accounted for.

## Existing genomic methods improve when context is considered

Despite the drawbacks of often defining pathogenicity as a binary and immutable feature of variants, genetic researchers have created many techniques of great utility. For example, molecular algorithms have been developed that can predict loss of protein function and these have high value in many settings^42–44^. We also now have protocols for molecular and clinical validation with laboratory-based functional assays^45^, and the longitudinal tracking of sequenced individuals in electronic health records^46^. Furthermore, several key papers have improved our thinking about the necessity of using diverse convergent evidence for causal reasoning in genomics^31,47,48^. Perhaps the most impressive advance in this area, is the scoring system developed by ClinGen that assembles and interprets empirical evidence for pathogenicity^49^. However, these approaches can only do so much when context is not explicitly considered. For example, even if we could develop a prediction algorithm that perfectly determined loss-of-function in any protein, we would still not know if loss-of-function was good or bad for any individual (given the remainder of their genome, and their environment, and the phenotype in question)^50–56^. Take for instance a protein that can convert pro-carcinogenic compounds to carcinogens. Loss-of-function of this protein may be beneficial in the context of high procarcinogen exposure^57^. Hence, the context, in this case the environment, can change a variant from beneficial to pathogenic and vice versa.

Therefore, even if we are using the best methods, we can observe conflicting evidence of pathogenicity when we do not explicitly consider context. This is particularly relevant for common variants. If a given variant is detrimental in all contexts, then this variant will usually be observed as a rare or de novo variant. In other words, variants are persistently culled by evolution when they reduce reproductive fitness in all contexts, but they can be maintained in the contexts where they do not reduce reproductive fitness. This may be especially evident when we consider pleiotropy, because antagonistic pleiotropy appears to play a major role in the persistence of several human disease variants^58,59^. For example, the strongest genetic determinant of Alzheimer’s Disease, APOE4^60,61^, also prevents death from diarrhea in childhood^62,63^. Our ancestors probably needed infection protection for their reproductive fitness and one of the variants that met this early life requirement, also increased the risk of a late life disease, Alzheimer’s Disease^62–66^. Thus, it makes very little sense to talk about the universal pathogenicity of any common variant. However, from a practical perspective, it is hard to do anything else.

## Context is complex—how can we specify it?

Context is easy to invoke as a concept, but the relevant context or determinants of penetrance, can differ for virtually every variant. Thus, when operationalizing research questions: What contexts do we measure? What contexts do we analyze? What phenotype do we examine? Even in the simplest research case with a single SNP, the potentially relevant context can be a cryptic and computationally impractical search space. Unfortunately, this explodes into intractability when considering Genome Wide Association or Next Generation Sequencing data (millions of SNPs and potentially thousands of environmental exposome variables). So, how can this problem be addressed? How can contexts that need attention be identified? It may be most practical to start with common and easily measured “contexts” that are known to have strong biological functions. This will help to optimize precision, statistical power, and the likelihood of documenting context-dependent pathogenicity.

With these features in mind, biological sex is among the easiest contexts to evaluate. It is easily measurable, it divides all human populations approximately in half, and there are many anatomic, physiologic, and pathophysiologic distinctions that align with it. Thus we can, and probably should, run sex-stratified sensitivity analyses in most genetic research studies^67–69^ especially when a trait is sexually dimorphic^70^. Failure to do this can obscure important biological patterns. Another step would be to encourage new methods for probing the X-chromosome, a chromosome that is often-ignored in association analyses. We have already started this strategy by analyzing the female-to-male allele frequency ratio as tool for the discovery of pathogenic variants (Equation 1)^71^. The reasoning is as follows: females have 2 copies of all Non-Pseudoautosomal X-chromosome loci and males only have one. Thus, females can be biologically more resilient to the presence of harmful variants at these sites. The exception is variants with dominant effects, in which case ratios will not be useful for detecting these variants. In any dataset of adult humans, when a Non-Pseudoautosomal X-chromosome variant exists at a higher proportion in females, this pattern can serve as evidence that the variant may increase the probability of premature death.

Following this simple logic, we used gnomAD data^72^ to characterize this phenomenon. Our methods are fully described in^71^, but in short, we obtained exome data from the X-Chromosomes of 76,702 males and 64,754 females. Then, we calculated female-to-male allele frequency ratios for the 44,606 variants that had an allele count of at least 5. None of the pseudoautosomal variants had a ratio above 11, but 319 of the non-pseudoautosomal variants had ratios above this empiric threshold.

Only 25 of these high-ratio variants were annotated in ClinVAR and had a rs number. Most of these variants had high sex-averaged MAFs and no known associations with disease, and they were listed as *benign* or *likely benign* (Table 1). As an example, one of the 25 variants had a sex-averaged MAF of 0.13, no known disease associations, and was listed as *likely benign*. This site had been genotyped 38,527 times in males (one locus each) and 104,056 times in females (2 loci each), so there was no shortage of data. Overall, the variant was observed a total of 18,736 times, but not one of these observations came from a male or a homozygous female. It was only found in heterozygous females. Thus, it is likely that this variant is almost 100% lethal (perhaps even embryonic lethal) in males and homozygous females, but is without large effect in heterozygous females. When we considered the other 24 variants, we found similar patterns, although the comparisons were less extreme.

**Table 1:** Variants identified in gnomAD exome data that have an allele proportion ratio above 11 and a ClinVar entry.

rs number and gene from dbSNP^73^	ClinVar entry^20,81^	Female to male allele proportion ratio^a^^71^	Listed as failing QC in the gnomAD Genome data^74^	Total no. of observations in the gnomAD Exome data^72,74^	Varsome pathogenicity scores summary^74^	PhyloP 100way Conservation score listed in varsome (higher = more conserved)^74^	Complications in interpretation noted in varsome^74^	Unique feature observed in varsome^74^	Gene- phenotype association from OMIM^75^	Predicted effect of variant on protein structure from Michelangelo – VENUS^76,77^
rs201580891 FMR1	Likely benign	6937.5	yes	18,736	1 Pathogenic 8 Uncertain 6 Benign	2.471	NA	Only observed in heterozygous females	Missense variant in FMR1—the gene linked to Fragile X Syndrome, Fragile X tremor/ataxia syndrome, Premature ovarian failure 1	Structurally neutral—K119N (a variant may be structurally neutral, but phenotypically deleterious)
rs1315062158 IQSEC	Benign/likely benign	1809.3	yes	3617	no data	0.985	NA	Only observed in heterozygous females	Synonymous variant in IQSEC – a gene linked X-linked intellectual developmental disorder-1	N/A Synonymous Variant
rs782666190 SMC1A	Benign	929.4	yes	4418	no data	0.100	NA	Males are rare, and homozygous females are absent	Intronic Variant in SMC1A – a gene tied to Cornelia de Lange syndrome-2, and developmental and epileptic encephalopathy-85	N/A Intronic Variant
rs1432363549 SMC1A	Likely benign	487.7	yes	968	no data	−0.691	NA	Only observed in heterozygous females	Intronic Variant in SMC1A – a gene tied to Cornelia de Lange syndrome-2, and developmental and epileptic encephalopathy-85	N/A Intronic Variant
rs782705493 HDAC8	Benign	379.3	yes	1657	no data	0.131	NA	Males and homozygous females are rare	Intronic Variant in HDAC8—a gene tied to Cornelia de Lange syndrome-5	N/A Intronic Variant
rs777010333 COL4A6	Likely benign	279.1	yes	563	no data	−0.995	NA	Only observed in heterozygous females	Intronic Variant in COL4A6—tied to X-linked deafness-6	N/A Intronic Variant
rs782664878 SMC1A	Benign	263.8	yes	8386	no data	−0.522	NA	Males and homozygous females are rare	Intronic Variant in SMC1A – a gene tied to Cornelia de Lange syndrome-2, and developmental and epileptic encephalopathy-85	N/A Intronic Variant
rs372580592 SLC9A6	Likely benign	223.1	yes	493	6 Benign	−0.104	NA	Only observed in heterozygous females	Intronic Variant in SLC9A6 – a gene tied to Christianson type of X-linked syndromic intellectual developmental disorder	N/A Intronic Variant
rs782792601 NDUFB11	Benign	201.2	yes	624	4 Pathogenic 1 Uncertain 2 Benign	1.656	NA	Males are absent and homozygous females are rare	Splice Donor Variant in NDUFB11—a gene tied to Linear skin defects with multiple congenital anomalies 3	N/A Splice Donor Variant (VENUS requires the specification of a single AA change at a single site)
rs782032695 EBP	Likely benign	194.5	yes	398	no data	0.342	NA	Only observed in heterozygous females	5’ UTR Variant in EBP—a gene tied to X-linked dominant chondrodysplasia punctata-2 and MEND syndrome	N/A 5’ UTR Variant
rs781824575 HDAC8	Benign	131.5	yes	6367	no data	−1.565	NA	Hemizygous Males and homozygous females are rare	Intronic Variant in HDAC8—a gene tied to Cornelia de Lange syndrome-5	N/A Intronic Variant
rs745354475 USP9X	Benign	129.4	yes	432	6 Benign	2.378	In a segmental duplication region	Only observed in heterozygous females	Intronic variant in USP9X – a gene tied to X-linked intellectual developmental disorder-99 and Female-restricted X-linked syndromic intellectual developmental disorder-99	N/A Intronic Variant
rs199626569 GJB1	Uncertain significance	89.3	NA	176	10 Pathogenic 8 Uncertain 4 Benign	8.015	NA	Only observed in heterozygous females	Missense variant in GJB1 – a gene tied to X-linked dominant Charcot-Marie-Tooth neuropathy 1	Structurally neutral—V166G (a variant may be structurally neutral, but phenotypically deleterious)
rs782072345 NDUFB11	Benign	87.6	no	1340	1 Pathogenic 1 Uncertain	1.254	NA	Hemizygous Males and homozygous females are rare	Intronic Variant in NDUFB11—a gene tied to Linear skin defects with multiple congenital anomalies 3	N/A Intronic Variant
rs745338783 POLA1	Likely benign	87.1	yes	201	10 Benign	−0.413	In a low complexity region	Only observed in heterozygous females	Intronic Variant in POLA1 – a gene tied to X-linked reticulate pigmentary disorder (PDR) with systemic manifestations and Van Esch-O’Driscoll syndrome	N/A Intronic Variant
rs751314374 RPGR	Conflicting (Uncertain significance and Benign)	70.7	yes	308	3 Uncertain 18 Benign	−1.198	In a low complexity region	Only observed in heterozygous females (more common in East and South Asians)	Missense Variant in RPGR—a gene tied to X-linked cone-rod dystrophy-1, X-linked atrophic macular degeneration, retinitis pigmentosa-3, and X-linked retinitis pigmentosa and sinorespiratory infections, with or without deafness	Structurally neutral - E934G (a variant may be structurally neutral, but phenotypically deleterious) This was run on the only isoform in VENUS that has at least 934 residues (Q92834)
rs1250133030 RPGR	Likely benign	64.9	yes	318	2 Uncertain 24 Benign	−0.145	In a low complexity region	Only observed in heterozygous females	Missense Variant in RPGR—a gene tied to X-linked cone-rod dystrophy-1, X-linked atrophic macular degeneration, retinitis pigmentosa-3, and X-linked retinitis pigmentosa and sinorespiratory infections, with or without deafness	Unclear - ClinVar lists this variant as creating a K857E amino acid change - only one isoform listed in VENUS has this many residues, and this isoform has an E at position 857
rs72609545 VCX3A	Benign	58.1	yes	1,218	2 Uncertain 21 Benign	−4.362	In a segmental duplication region	Hemizygous Males and homozygous females are rare	Missense Variant in VCX3A – a gene putatively tied to X-linked Ichthyosis (it is in a region that is implicated)	Structurally neutral - V140M (a variant may be structurally neutral, but phenotypically deleterious)
rs12849277 MED12	Benign/Likely benign	55.2	yes	83	No data	−1.295	In a low complexity region	Only observed in heterozygous females	Intronic variant in MED12 – a gene tied to Hardikar syndrome, Lujan-Fryns syndrome, X-linked Ohdo syndrome, and Opitz-Kaveggia syndrome	N/A Intronic Variant
rs781379769 USP9X	Benign/Likely Benign	53.3	yes	6602	2 Benign	−0.241	In a low complexity region	Hemizygous Males and homozygous females are rare	Intronic variant in USP9X – a gene tied to X-linked intellectual developmental disorder-99 and Female-restricted X-linked syndromic intellectual developmental disorder-99	N/A Intronic Variant
rs145404090 SAGE1	Uncertain significance	44.8	yes	79	3 Uncertain 23 Benign	0.252	In a segmental duplication region	Only observed in heterozygous females 92% were European	Missense variant in SAGE1 – a geneputatively tied to cancer Part of a set of genes that are only expressed in tumors, spermatogenic and placental cells	Structurally neutral—T203A (a variant may be structurally neutral, but phenotypically deleterious)
rs148934011 RBMX	Uncertain significance	33.6	yes	56	5 Pathogenic 8 Uncertain 2 Benign	6.024	In a segmental duplication region	Only observed in heterozygous females 95% were African	Missense variant in RBMX – a gene tied to X-linked syndromic intellectual developmental disorder-11, Sashi type	Stabilizing - D333Y (a variant may be stabilizing, but phenotypically deleterious)
rs782233695 RHOXF2	Likely benign	22.7	yes	39	4 Uncertain 24 Benign	−4.628	In a segmental duplication region	Only observed in heterozygous females 97% were South Asian	Missense variant in RHOXF2 – a gene with no phenotypes noted in OMIM	Unclear - ClinVar notes that the change is H139Y, but VENUS notes there is an N at position 139
rs1446705794 RPGR	Likely benign	20.9	yes	91	no data	−1.556	In a low complexity region	Only observed in heterozygous females	Synonymous Variant in RPGR—a gene tied to X-linked cone-rod dystrophy-1, X-linked atrophic macular degeneration, retinitis pigmentosa-3, and X-linked retinitis pigmentosa and sinorespiratory infections, with or without deafness	N/A Synonymous Variant
rs201558029 DMD	Benign	15.7	no	26	no data	1.541	NA	Only observed in heterozygous African or Latino Females	Intronic Variant in DMD – a gene linked to Becker muscular dystrophy, Duchenne muscular dystrophy, and X-linked dilated cardiomyopathy-3B	N/A Intronic Variant

^a^The female to male allele proportion ratio: $\,{\boldsymbol{R}}=\frac{({{\rm{V}}}_{f}+{\bf{1}})/({A}_{f}+{\bf{1}})}{({{\rm{V}}}_{m}+{\bf{1}})/({A}_{m}+{\bf{1}})}$

*R* allele proportion ratio, *V*_f_ the minor allele count in females, *A*_f_ the total allele count in females, *V*_m_ the minor allele count in males, *A*_m_ the total allele count in males. Note that 1 is added to the numerators and denominators to avoid dividing by 0. This allows a female-to-male allele proportion ratio to be calculated when no male carriers are observed with a given variant.

To further characterize these variants, we probed them with a diverse set of web-based bioinformatic resources: dbSNP^73^, VarSome^74^, OMIM^75^, and VENUS^76,77^. These databases provide additional information on evolutionary conservation, gene-phenotype relationships, protein-structure predictions, and other aspects of these variants that need consideration in pathogenicity assessments. We found that:

1. Existing annotation methods can miss sex-specific pathogenicity. We observed that 22 out of 25 (88%) high ratio variants are listed as Benign or Likely Benign in ClinVar (1 is listed as Conflicting [Uncertain Significance and Benign] 2 are listed as Uncertain Significance). These variants are commonly observed in healthy heterozygous females and they achieve high sex-averaged MAFs so they appear benign, but males are rarely observed (i.e., these variants are not often tolerated in males)
2. QC procedures can mislabel evidence of sex-specific pathogenicity as genotyping error. We looked in the second dataset from gnomAD site (the genomes data) and observed that 22 out of the 25 (88%) high ratio variants failed QC filters^74^. Sex differences in MAF were assumed to be error rather than putative evidence of sex-specific pathogenicity. Thus, these QC filters may systematically remove variants with sex-specific pathogenicity before they can even be assessed.
3. Our ratio method identified genes that were already linked to clinical syndromes through other variants. In all, 23 of 25 (92%) genes implicated by the high ratio variants have specific links to clinical syndromes listed in OMIM^75^. The other two genes have tentative links to pathology described in their OMIM entry.
4. Structural predictions are not available or useful for most of these top ratio hits. Michaelangelo-VENUS structural predictions^76,77^ were only possible for 6 of the 25 variants (24%). VENUS requires the specification of a specific amino acid substitution at a specific site in the protein. This makes sense for some variants, but 19 of the 25 variants do not have that impact, or their exact impact on amino acid sequence cannot be yet specified (synonymous, intronic, splice donor variants, etc.)
5. Additional heterogeneity exists and some high ratio variants might be better tolerated by males and homozygous females in specific contexts. Some high ratio alleles had frequencies that differed by ancestry group, and this is consistent with the interpretation that these variants may not have sex-specific pathogenicity in all contexts.

Overall, these 5 points indicate that seeking and documenting evidence of sex-specific effects could improve pathogenicity annotations. The existing tools for variant characterization can only do so much if context is not explicitly evaluated. Finally, we note that the many potential mechanisms for sex-specific pathogenicity remain to be characterized, but there is some indication in our initial results that regulatory function may sometimes be involved. RegulomeDB evaluations of the 25 high-ratio variants provide diverse and nuanced information on the likelihood of regulatory function at these loci (Table 2). They reveal that 13 of the 25 high ratio variants (52%) have some indication of regulatory function: a rank less than three or a score greater than 0.5. A rank less than three indicates the presence of at least two strong pieces of experimental evidence that are consistent with regulatory function, and scores greater than 0.5 are in the top half of possible scores from models that predict transcription factor binding.

**Table 2:** Evidence of regulatory function among the high ratio variants.

rs number from dbSNP^73^	RegulomeDB—rank^82,83^ ^1^ Integrative metric based on existing evidence 1 = strong evidence 7 = no evidence	RegulomeDB—score^82,83^ prediction based on transcription factor binding models 0 = lowest probability of regulatory function 1 = highest probability of regulatory function
rs201580891	7	0.18412
rs1315062158	5	0.38000
rs782666190	5	0.00454
rs1432363549	5	0.00000
rs782705493	2b	0.73553
rs777010333	5	0.01895
rs782664878	5	0.00000
rs372580592	5	0.09659
rs782792601	4	0.70497
rs782032695	2b	0.48000
rs781824575	2b	0.79371
rs745354475	5	0.00000
rs199626569	4	0.60906
rs782072345	4	0.70497
rs745338783	5	0.00125
rs751314374	5	0.58955
rs1250133030	5	0.58955
rs72609545	5	0.58955
rs12849277	5	0.58955
rs781379769	5	0.00000
rs145404090	5	0.23589
rs148934011	5	0.58955
rs782233695	4	0.60906
rs1446705794	5	0.98500
rs201558029	7	0.18412

^1^Possible scores in the RegulomeDB ranking system.

1a eQTL/caQTL + TF binding + matched TF motif + matched Footprint + chromatin accessibility peak.

1b eQTL/caQTL + TF binding + any motif + Footprint + chromatin accessibility peak.

1c eQTL/caQTL + TF binding + matched TF motif + chromatin accessibility peak.

1d eQTL/caQTL + TF binding + any motif + chromatin accessibility peak.

1e eQTL/caQTL + TF binding + matched TF motif.

1f eQTL/caQTL + TF binding / chromatin accessibility peak.

2a TF binding + matched TF motif + matched Footprint + chromatin accessibility peak.

2b TF binding + any motif + Footprint + chromatin accessibility peak.

2c TF binding + matched TF motif + chromatin accessibility peak.

3a TF binding + any motif + chromatin accessibility peak.

3b TF binding + matched TF motif.

4 TF binding + chromatin accessibility peak.

5 TF binding or chromatin accessibility peak.

6 Motif hit.

7 Other.

Sex differences in allele frequency on the X chromosome are a special case, but this pattern may also be found in autosomal variants that affect disease risk differently between males and females. Very large and very small allele proportion ratios in the autosomes may also be indicative of sex-specific effects that deserve further investigation. While this area of genetic research is still in its infancy, and thresholds for discovery and confirmatory findings are not yet established, we have already observed extreme female-to-male allele proportion ratios on autosomes (many standard deviations above or below the mean). Work in progress has already revealed a distribution of ratios on chromosome 21 that demonstrates this point (Table 3). Ratios this high are very unlikely occur by chance. Finally, we note that biological sex is just the first and simplest context to consider. More complex situations such as ancestry and environmental exposures will need increased attention. For example, we already know that failing to assess ancestry-specific associations can generate ancestry-specific misinterpretations of genetic tests that disproportionally harm marginalized groups^78^. We need to collect genetic data on diverse ancestry groups^79^ and explicitly consider this context in order to avoid generating health disparities with ancestry-specific medical error^80^.

**Table 3:** Summary statistics for the 21493 female-to-male allele proportion ratios calculated on chromosome 21 in the GnomAD exomes data.

	Mean	SD	Min	Max
Ratio	1.5	1.1	0.1	43.6
Log2(Ratio)	0.3	0.8	−2.7	5.4

Overall, considering context will not solve all the problems in pathogenicity assessment, but it is a necessary step for addressing key clinical and translational issues in genetics. Sex-stratified GWAS^70^, and female-to-male allele proportion ratios^71^ can start us on a path that probes multiple determinants of penetrance. A lot of work remains in determining how to best explore contextual frameworks for variant pathogenicity, and other tools will be needed to evaluate additional factors, such as xenobiotic exposures and ancestry. However, biological sex is an ideal context to start with, because it will not require any new data. Information on biological sex is extractable from virtually all existing genomic data, and these data can be easily re-evaluated at low cost. Furthermore, it will not be hard or expensive to better evaluate sex differentials in allele frequency and improve the definition of *benign* in pathogenicity annotations. As an easy first step, ClinVar could present MAFs by sex. Overall, we call on the genetic research community to proactively consider context. While the optimal frameworks for achieving this goal are not fully established, we can to start by routinely evaluating the sexes separately, and documenting what is known about effect modifiers in our annotations. We have proposed a deeper dive into sex as a common effect modifier but other strata should be explored and documented in annotations. Covariates should be collected in our datasets and exploratory sensitivity analyses should be more routine or we will fail to identify many determinants of penetrance that have clinical relevance.

## Conclusion

In summary, these strategies will not provide better answers to the old questions; they simply refine the questions so that they are more relevant. The old questions are generally context agnostic, and they have set the basis of our understanding reasonably well, but not well enough. If we want to keep advancing, we must now address the ubiquity of pleiotropy and the contextual determinants of penetrance.

Equation 1. The female-to-male allele proportion ratio^71^

$${\boldsymbol{R}}=\frac{({{\rm{V}}}_{\rm{f}}+{\bf{1}})/({A}_{\rm{f}}+{\bf{1}})}{({{\rm{V}}}_{\rm{m}}+{\bf{1}})/({A}_{\rm{m}}+{\bf{1}})}$$

*R*: allele proportion ratio

*V*_f_: the minor allele count in females

*A*_f_: the total allele count in females

*V*_m_: the minor allele count in males

*A*_m_: the total allele count in males

### Supplementary information

Supplemental Materials

## Data Availability

All data are public and available from https://gnomad.broadinstitute.org/.

## Code availability

The data handling and ratio calculations are previously described^71^. We re-evaluated the hits in publicly available web databases, but there is no new code associated with this commentary.
