# Supplementary material for: Characterizing the pathogenicity of genetic variants: the consequences of context
Source: NPJ Genom Med. 2024 Jan 9;9:3. doi: 10.1038/s41525-023-00386-5 (PMC10776585; doi:10.1038/s41525-023-00386-5)
Supplement: Supplementary file 1 — Supplemental Materials [file 41525_2023_386_MOESM1_ESM.docx]

**Supplemental Materials**

**Characterizing the pathogenicity of genetic variants: the consequences of context**

Our methods are fully described in ^1^, but in short, we downloaded exome data from the X-Chromosomes of 76,702 males and 64,754 females in the Genome Aggregation Database (gnomAD; https://gnomad.broadinstitute.org/) on June 17, 2020 (File: V2—GRCh37/hg19 referencesequence Gnomad.exomes.r2.1.1.sites.X.vcf.bg).^2^ This is an aggregated collection of exome data from multiple studies and ancestry groups, and the full details of data cleaning and preparation are available from ^2^. The file contained 348,221 X-chromosome variants that cleared gnomAD quality control procedures. For our analyses we removed loci with duplicate entries (n=52,583) and rare variants (Minor Allele Count [MAC]<5 in females; n=251,032). Then, using equation 1, we calculated female-to-male allele frequency ratios for the remaining 44,606 variants. None of the pseudoautosomal variants had a ratio above 11, but 319 of the non-pseudoautosomal variants had ratios above this empiric threshold. Only 25 of these high-ratio variants had rs numbers and were annotated in ClinVAR (see Table 1 in the main text).

__________________________________________________________________________________________

Equation 1. The female-to-male allele proportion ratio ^1^

$$\boldsymbol{R=}\frac{\boldsymbol{(}V_{f}\boldsymbol{+1)/(}A_{f}\boldsymbol{+1)}}{\boldsymbol{(}V_{m}\boldsymbol{+1)/(}A_{m}\boldsymbol{+1)}}$$

R: allele proportion ratio

V_f_: the minor allele count in females

A_f_: the total allele count in females

V_m_: the minor allele count in males

A_m_: the total allele count in males

___________________________________________________________________________________

**References**

1. Ciesielski, T. H., Bartlett, J., Iyengar, S. K. & Williams, S. M. Hemizygosity can reveal variant pathogenicity on the X-chromosome. *Hum Genet* **142**, 11–19 (2023).

2. Karczewski, K. J. *et al.* The mutational constraint spectrum quantified from variation in 141,456 humans. *Nature* **581**, 434–443 (2020).
